# Letter to the Editor Re: Teoh SL *et al.*, *Nutrients* 2016, 8, 57

**DOI:** 10.3390/nu8040201

**Published:** 2016-04-06

**Authors:** Morgan E. Stewart

**Affiliations:** Morgan Stewart Consulting, 3669 Lake Park Rd., Fallbrook, CA 92028, USA; morgan_stewart@mindspring.com; Tel.: +1-760-451-6080

Dear Editor,

We read with interest the recently published meta-analysis report titled “Chicken Essence for Cognitive Function Improvement: A Systematic Review and Meta-Analysis” by Teoh *et al.* [1] in *Nutrients*. We feel that a substantial number of questions are raised by this analysis, none of which are sufficiently addressed by the authors. The questions and issues can be grouped into general categories, and these are given below in order of decreasing importance.

## 1. Methodological and External Validity Issues

Reference 2 (Azhar *et al.*, 2013 [2]) was a study on CMI-168 (as described in the abstract), not Chicken Essence, and thus this study was inappropriate for inclusion.

Of the studies considered for inclusion, a very large number had to be eliminated. While the grounds for inclusion in the combined analysis were very broad, of 2203 non-duplicate studies reviewed, only seven were found to be suitable for qualitative synthesis and five for meta-analysis, and the total number of included subjects (363) was relatively small. In four of these studies there was either an unacceptably high dropout rate (>20%) or the dropout rate was not stated.

Fifty-six of the subjects came from a single study of patients with poor cognitive function (Reference 14 (Azhar *et al.*, 2003 [3]), while the remainder of the subjects came from populations of healthy volunteers. In our view, combining cognitively impaired subjects’ results with those of healthy volunteers is not valid; grouping these subjects together introduces the potential for bias, as possible outliers may neutralize any statistically or clinically significant results. In addition, five of the studies used a narrow age range of young subjects (<40 years)—these subjects are less likely to show a cognitive effect even if one existed.

The multiplicity of endpoints in the seven studies made it impractical to measure anything consistently across all the studies. For example, Azhar *et al.* [2] used in the meta-analysis measured long-term memory, while in the other studies, aspects of short-term memory, working memory, attention, *etc.*, were measured. Study designs also differed, as four of the studies were parallel and three were crossover.

The product types, formats, treatment periods, and dosages were extremely heterogeneous across the seven studies.

The authors attempted to establish a connection between Chicken Essence, which includes a wide variety of peptides, and cognitive function. In the Discussion section, the authors compare the results of Chicken Essence studies to studies using carnosine. While carnosine is one of the many peptides that can be found in the Chicken Essence, the Chicken Essence products contain hundreds of different peptides with various activity levels; in addition, carnosine makes up less than 1/1000 fraction of Chicken Essence solid content. Because of these factors and the differing formulations of products used in the studies, the effect of carnosine on cognitive function and its mechanism of action cannot be determined. Therefore, there is no rationale to link Chicken Essence studies to carnosine studies as the authors have done.

“Checking” methods (e.g., Cochran’s Risk of Bias) applied to the various studies determined that the individual study results were, *a priori*, unreliable. We believe that it does not make sense to combine a small group of poorly done studies in a meta-analysis.

## 2. Statistical Issues

Thirty-five significance tests of mean differences were conducted and twelve were significant at the 0.05 level. While the significant outcomes tended to group together (in Executive Function and Short Term Memory, as one would expect if these assessments are intercorrelated), the actual tests were mostly different in the different studies, making comparisons difficult.

## 3. Accuracy Issues

Table 4 identifies Executive Function, WAIS arithmetic test, Azhar *et al.*, 2008 [4], as significant, but the 95% CI includes zero.

## 4. Conclusions

While the authors concluded that a claim of cognitive benefit for Chicken Essence was not yet supported by published data, the very large number of methodological issues identified above leads us to believe that, at this point in time, evidence produced in this paper are too scanty and potentially unreliable to permit any conclusion, either positive or negative, to be drawn. To assess the impact of Chicken Essence on cognitive function through a meta-analysis, future research needs to identify studies that are consistent across product formulation, dosage, and treatment duration, use subjects in sufficient numbers drawn from comparable populations, and have clear, homogeneous cognitive endpoints.
